# Pandemic influenza and subsequent risk of type 1 diabetes: a nationwide cohort study

**DOI:** 10.1007/s00125-018-4662-7

**Published:** 2018-06-22

**Authors:** Paz L. D. Ruiz, German Tapia, Inger J. Bakken, Siri E. Håberg, Olav Hungnes, Hanne L. Gulseth, Lars C. Stene

**Affiliations:** 1 Department of Chronic Diseases and Ageing, Norwegian Institute of Public Health, Postbox 4404, Nydalen, 0403 Oslo, Norway; 2 Department of Endocrinology, Morbid Obesity and Preventive Medicine, Oslo University Hospital, Oslo, Norway; 3 Institute of Clinical Medicine, University of Oslo, Oslo, Norway; 4 Centre for Fertility and Health, Norwegian Institute of Public Health, Oslo, Norway; 5 Department of Influenza, Norwegian Institute of Public Health, Oslo, Norway

**Keywords:** Incidence, Infections, Influenza, Influenza H1N1, Register-based study, Type 1 diabetes

## Abstract

**Aims/hypothesis:**

Case reports have linked influenza infections to the development of type 1 diabetes. We investigated whether pandemic and seasonal influenza infections were associated with subsequent increased risk of type 1 diabetes.

**Methods:**

In this population-based registry study, we linked individual-level data from national health registries for the entire Norwegian population under the age of 30 years for the years 2006–2014 (2.5 million individuals). Data were obtained from the National Registry (population data), the Norwegian Patient Registry (data on inpatient and outpatient specialist care), the Primary Care Database, the Norwegian Prescription Database and the Norwegian Surveillance System for Communicable Diseases. Pandemic influenza was defined as either a clinical influenza diagnosis during the main pandemic period or a laboratory-confirmed test. Seasonal influenza was defined by a clinical diagnosis of influenza between 2006 and 2014. We used Cox regression to estimate HRs for new-onset type 1 diabetes after an influenza infection, adjusted for year of birth, sex, place of birth and education.

**Results:**

The adjusted HR for type 1 diabetes after pandemic influenza infection was 1.19 (95% CI 0.97, 1.46). In the subgroup with laboratory-confirmed influenza A (H1N1), influenza was associated with a twofold higher risk of subsequent type 1 diabetes before age 30 years (adjusted HR: 2.26, 95% CI 1.51, 3.38).

**Conclusions/interpretation:**

Overall, we could not demonstrate a clear association between clinically reported pandemic influenza infection and incident type 1 diabetes. However, we found a twofold excess of incident diabetes in the subgroup with laboratory-confirmed pandemic influenza A (H1N1).

**Electronic supplementary material:**

The online version of this article (10.1007/s00125-018-4662-7) contains peer-reviewed but unedited supplementary material, which is available to authorised users.

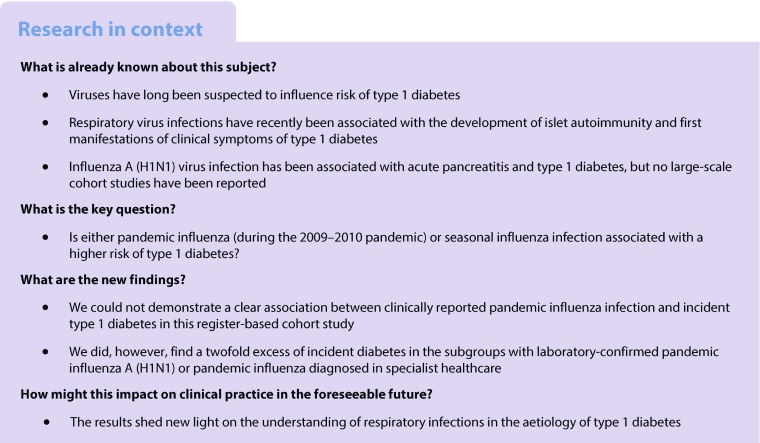



## Introduction

Type 1 diabetes is a chronic autoimmune disease with both genetic and environmental contributions. Viruses may influence susceptibility and trigger autoimmunity in individuals genetically predisposed to diabetes [1, 2]. Enteroviruses and other viruses have been most frequently studied in relation to type 1 diabetes [3]. Recently, respiratory virus infections have also been associated with the development of islet autoimmunity and the first manifestations of clinical symptoms of type 1 diabetes [4–8]. Influenza virus can infect human pancreatic cell lines, and can cause pancreatitis and hyperglycaemia in animal models [9]. Influenza A (H1N1) virus infection has also been associated with acute pancreatitis [10–12] and type 1 diabetes [13, 14] in human case-report studies.

Influenza spreads yearly across the continents, and when a new influenza virus emerges and transmits among humans, an influenza pandemic can occur [15]. In April 2009, the World Health Organization detected an outbreak of a new influenza virus [Influenza A(H1N1)pdm09] in Mexico. This influenza, the swine flu, was declared a pandemic in June 2009, and this lasted until August 2010 [16]. The virus has subsequently continued to circulate as one of the seasonal influenza strains.

Two small retrospective studies have suggested that pandemic influenza may be associated with type 1 diabetes [17, 18]. These showed a concomitant increase in type 1 diabetes during the pandemic influenza period among children. However, as the typical time from induction of islet autoimmunity to clinical onset of type 1 diabetes is several years [19], studies with longer follow-up after influenza are necessary to elucidate the role of pandemic influenza in development of diabetes.

No previously published studies have addressed whether pandemic influenza diagnosis is associated with the development of type 1 diabetes. We have recently reported that there was no association between vaccination against the 2009 H1N1 pandemic influenza virus with the AS03 adjuvanted Pandemrix vaccine and type 1 diabetes [20]. In Norway, the vaccination campaign and the main influenza wave occurred simultaneously [21, 22]. Here, we investigate a potential relationship between pandemic or seasonal influenza in the years 2006–2014 and subsequent type 1 diabetes in a nationwide register-based cohort study from Norway.

## Methods

### Participants and design

**Fig. 1:**
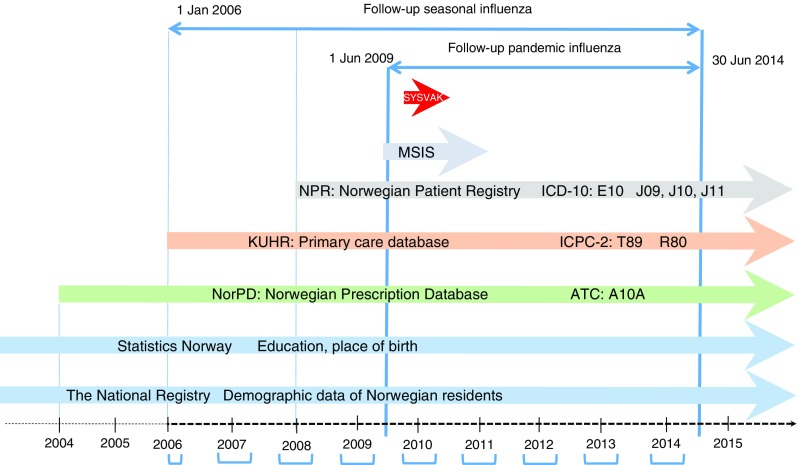
Overview of nationwide registers linked via personal identification numbers assigned to all Norwegian residents. Laboratory-confirmed influenza data are from the MSIS register, May 2009 to April 2011. The Norwegian Patient Registry collects data on individuals receiving specialist healthcare (inpatient and outpatient) (see ESM Table 1 for codes and abbreviations). SYSVAK is a national electronic immunisation registry that records an individual’s vaccination status and vaccination coverage in Norway (ICD-10, ICPC-2, the Anatomical Therapeutic Chemical). Blue brackets represent the seasonal influenza period each year (for 2006, data were available from 1 January). ATC, Anatomical Therapeutic Chemical; Jan, January; Jun, June

In this open cohort study, we linked individual-level data from seven national registers with prospectively collected data for more than 2.5 million residents in Norway aged 30 years and younger, and followed them from 2006 to 30 June 2014 (Fig. 1).

The study population consisted of all residents in Norway as registered in the National Registry. Statistics Norway provided data on education and place of birth. Further, we obtained information on use of glucose-lowering drugs from the Norwegian Prescription Database, and diabetes diagnosis codes and dates of diagnoses from the Primary Care Database and the Norwegian Patient Registry. Laboratory-confirmed influenza cases were registered in the Norwegian Surveillance System for Communicable Diseases (MSIS). (Details of each register is outlined in the electronic supplementary material [ESM].) Information in each database is registered with the personal identification number that is given to all Norwegian residents, which enables linking on an individual level.

The study was approved by the Norwegian Data Protection Authority (approval number 10/00910-12) and the Regional Committee for Medical and Health Research Ethics (approval number 2010/2583).

### Diagnosis of type 1 diabetes before age 30 years

We defined incident cases of type 1 diabetes as registration of dispensed insulin for at least 6 months and at least one registration of a type 1 diabetes diagnosis from specialist or primary care (see ESM Table 1 for included classification codes and abbreviations). To ensure inclusion of type 1 diabetes only, we excluded individuals who received oral glucose-lowering agents within 1 year after diagnosis. To avoid any prevalent cases of diabetes at baseline, we excluded individuals who had a diagnosis of any type of diabetes or used any glucose-lowering medication before the start of the study (Fig. 1).

### Exposures

The primary exposure was pandemic influenza infection, and people were defined as having pandemic influenza either by a diagnosis of influenza registered in the national primary care database (using the International Classification of Primary Care, Second Edition [ICPC-2] code R80) or in the Norwegian Patient Registry (specialist care, coding according to the ICD-10: J09, J10, J11 [www.who.int/classifications/icd/en/]) during the pandemic period or by laboratory-confirmed pandemic influenza registered in the Norwegian Surveillance System for Communicable Diseases (ESM Table 1). We defined the pandemic influenza period as starting in June 2009 and lasting until May 2010.

The secondary exposure was seasonal influenza infection, defined as any influenza diagnosis occurring in the surveillance periods for influenza in Norway. We analysed each influenza season, from around October to mid-May each year, in the years 2006 to 2012–2013, with the exception of the 2009–2010 season, which was the pandemic influenza period defined as above. The pandemic season was included in the seasonal analysis.

### Subgroups/stratification

We additionally performed analyses separately for males and females, and for people under 15 years of age. We did subgroup analysis for those registered with a laboratory-confirmed influenza diagnosis, those registered in primary care and those registered in specialist care.

### Covariates

Information on sex, date of birth, emigration, immigration and death were obtained from the National Registry. Information on vaccination with Pandemrix, an AS03-adjuvanted influenza A(H1N1)pdm09 vaccine, was obtained from the Norwegian Immunisation Register [23], and Statistics Norway provided data on place of birth and education (Fig. 1). We used the highest education in year 2013 for the participant or his/her parents for all the individuals in the analyses. Place of birth was classified into three categories (‘Norway’, ‘Europe except Norway’ and ‘outside Europe’). Information about seasonal influenza vaccinations or laboratory-confirmed influenza outside the pandemic was not available.

### Sensitivity analysis

During the pandemic period (June 2009 until May 2010) other viruses may have caused influenza-like symptoms [24]. In a sensitivity analysis we restricted to the peak pandemic period, October 2009 to December 2009, where there were no other influenza viruses in circulation. The main influenza wave and the vaccination campaign occurred at the same time in Norway, and vaccinated individuals could also be registered with influenza diagnosis. In a sensitivity analysis we restricted to those who were not vaccinated against pandemic influenza.

### Statistical analyses

We used Cox regression with months as the time metric and influenza as time-dependent exposure variable to estimate HRs with 95% CI for type 1 diabetes, both unadjusted and adjusted for year of birth (in groups of 3 years), sex, place of birth and education. The study population was followed from birth, 1 year after immigration or start of follow-up (January 2006), whichever occurred last, until type 1 diabetes diagnosis, emigration, death, 30 years of age or end of follow-up (July 2014), whichever occurred first. Immigrants were included for follow-up 1 year after immigration to avoid prevalent cases of type 1 diabetes being misclassified as incident and to ensure that influenza exposure could be registered.

In the model where we estimated risk of type 1 diabetes after pandemic influenza, follow-up started in June 2009. We performed separate analyses for each influenza season, with start of follow-up in October each year for the same calendar year, except for the 2005–2006 season, when follow-up started in January 2006 (the beginning of our observation period). Influenza was included as time-varying exposure for which individuals contributed with unexposed person-time until their first month of influenza diagnosis, and were regarded as exposed afterwards.

Data handling and analyses were done using Stata version 14 (Stata Statistical Software: Release 14, StataCorp, College Station, TX, USA).

## Results

### Pandemic influenza and risk of type 1 diabetes

Among the 2,286,650 individuals in the study population, 76,173 (3.3%) were diagnosed with pandemic influenza. From June 2009 to December 2014, 2376 individuals (0.1%) were diagnosed with new-onset type 1 diabetes (Table 1). New-onset type 1 diabetes was registered at a slightly younger age for those registered with pandemic influenza diagnosis compared with those without such registration (12.9 years compared with 13.3 years).

**Table 1:** Characteristics of the study population aged <30 years in the pandemic analysis

Characteristics	Individuals		
	All *n* = 2,286,650	With pandemic influenza^a^ *n* = 76,173	With incident type 1 diabetes *n* = 2376
Sex			
Male	1,169,485 (51)	37,344 (49)	1382 (58)
Female	1,117,165 (49)	38,829 (51)	994 (42)
Year of birth			
1979–1989	713,963 (31)	25,563 (34)	339 (14)
1990–1999	665,198 (29)	24,904 (33)	934 (39)
2000–2009	628,606 (27)	25,694 (34)	1020 (43)
>2010	278,883 (12)	12 (0)	83 (4)
Education level^b^			
≤10 years	200,040 (9)	7838 (10)	165 (7)
11–13 years	779,682 (34)	30,289 (40)	953 (40)
≥14 years	1,214,276 (53)	37,076 (49)	1244 (52)
No information	92,652 (4)	970 (1)	15 (1)
Place of birth			
Norway	1,973,332 (86)	67,667 (89)	2260 (95)
Europe (except Norway)	166,130 (7)	3582 (5)	63 (3)
Outside Europe	147,188 (6)	4924 (6)	53 (2)

Data shown are *n* (%)

^a^See Methods for details of influenza diagnosis. Note that individuals with pandemic influenza and individuals with incident type 1 diabetes were not mutually exclusive (numbers in the two columns therefore do not add to the total)

^b^The highest education level the individual achieved up to 2013 or the highest attained education level of their parents

**Fig. 2:**
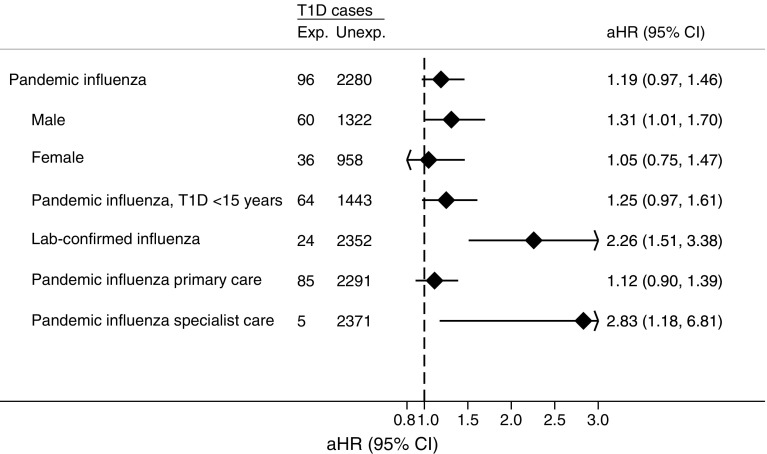
Association between pandemic influenza diagnosis and risk of type 1 diabetes in up to 2.28 million Norwegian residents under 30 years of age, overall and in subgroups. Incident cases of type 1 diabetes defined as registration of dispensed insulin for at least 6 months and at least one registration of a type 1 diabetes diagnosis from specialist or primary care. Pandemic influenza was defined as a clinical diagnosis of influenza registered in the primary care database, specialist care, or a laboratory-confirmed pandemic influenza (during the pandemic period). HRs were adjusted for year of birth, sex, place of birth, education and pandemic influenza vaccination (except analysis stratified for sex, which was adjusted for year of birth, place of birth, education and pandemic influenza vaccination). Exp., exposed; Lab, laboratory; T1D, type 1 diabetes; Unexp., unexposed

**Fig. 3:**
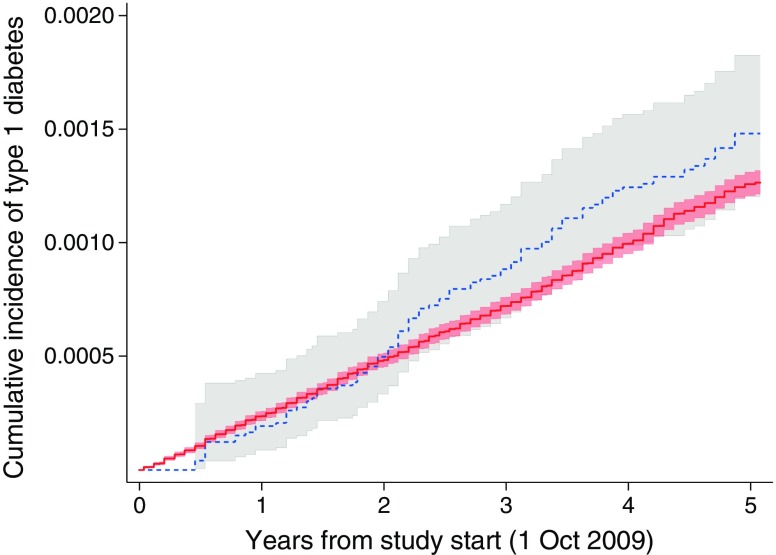
Cumulative incidence and 95% CI of type 1 diabetes for pandemic influenza (blue line and grey shaded area) and for no pandemic influenza (red line and light red shaded area). Logrank test *p* = 0.049

Pandemic influenza diagnosis was associated with an approximately 20% higher risk of type 1 diabetes, though this was not statistically significant (Fig. 2). The cumulative incidence of being diagnosed with type 1 diabetes during the study period for those with and without a registered influenza diagnosis is shown in Fig. 3. Among those diagnosed with influenza during the pandemic, 11.4% had laboratory-confirmed pandemic influenza (7.4% of influenza diagnoses in primary care and 48.4% of influenza diagnoses registered in specialist healthcare). When restricting analyses to those with a laboratory-confirmed pandemic influenza, there was a twofold higher risk of type 1 diabetes (adjusted HR 2.26, 95% CI 1.51, 3.38, Fig. 2). Those who developed type 1 diabetes after a laboratory-confirmed influenza A infection were, on average, 10 years old at the time of influenza, and were diagnosed with type 1 diabetes on average 2.2 years later (ESM Fig. 1). In the analysis of those with pandemic influenza diagnosed in specialist healthcare, the adjusted HR was 2.83, 95% CI 1.18, 6.81 (Fig. 2; of those, four of five new cases of type 1 diabetes were laboratory confirmed).

### Seasonal influenza and type 1 diabetes

**Fig. 4:**
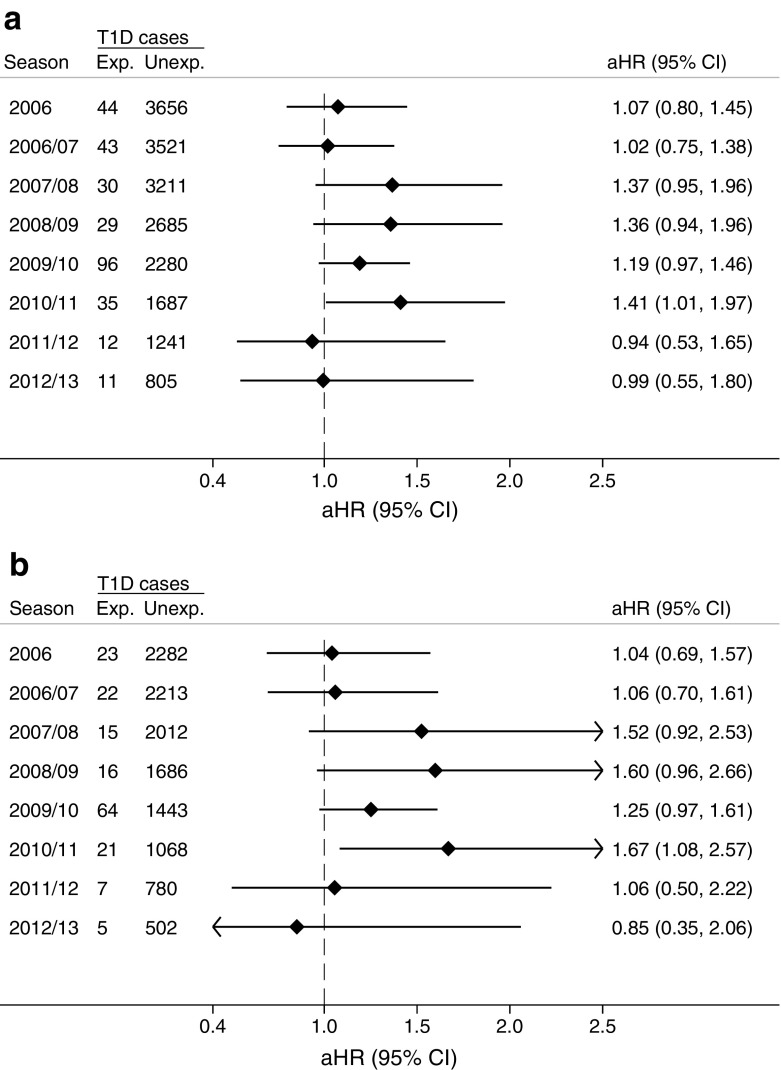
Association between seasonal influenza diagnosis and risk of type 1 diabetes in more than 2.5 million individuals under 30 years of age (**a**), and under 15 years of age (**b**). HRs were adjusted for year of birth, sex, place of birth and education. Seasonal influenza from 1 January 2006 to 30 June 2014 (the pandemic season, 2009–2010, shows the same data as in Fig. 2; during this season, pandemic influenza was defined as an influenza registration in primary care or in specialist care or laboratory-confirmed pandemic influenza). Exp., exposed; Unexp., unexposed

During the study period for seasonal influenza, from 2006 to mid-2014, 3700 individuals under 30 years of age were diagnosed with type 1 diabetes during 15,583,847 person-years of follow-up (including the 2009–2010 pandemic influenza period). The number of individuals registered with a seasonal influenza diagnosis included in the analysis varied from 19,691 in the season 2007–2008 to 39,179 in the 2012–2013 season (ESM Table 2 and ESM Fig. 2). Higher risk of type 1 diabetes after a seasonal influenza diagnosis was observed in all seasons between 2007 and 2011, but only the season 2010–2011 was statistically significantly associated in both the total population and those aged below 15 years (Fig. 4).

### Sensitivity analyses

We assessed whether the pandemic influenza association with type 1 diabetes was different when we restricted to influenza diagnoses made during the peak pandemic period which, in Norway, occurred from 1 October 2009 to 31 December 2009 [25]. This restriction resulted in a slightly smaller number of exposed individuals, but yielded very similar results (adjusted HR 1.17, 95% CI 0.95, 1.45).

Second, to be able to analyse the association of the different seasons of influenza and have similar follow-up times after infection (for better comparison of the association with type 1 diabetes across seasons), we defined the time-dependent exposure (seasonal influenza) to last for a maximum of 2 years after influenza diagnosis. For instance, a person exposed to seasonal influenza diagnosis in 2006 would be regarded as exposed in the following 2 years, but after 2008 would contribute unexposed person-time unless he/she developed type 1 diabetes before this time). These analyses gave similar results as the main analyses (ESM Fig. 3).

In the analyses with pandemic influenza as the exposure, we performed an analysis where we adjusted for vaccination with Pandemrix, and the results were not modified (adjusted HR [aHR] 1.19, 95% CI 0.97, 1.46 for all diagnosed with pandemic influenza). Finally, when we restricted the analyses to those who were not vaccinated against pandemic influenza, pandemic influenza was associated with an increased risk of type 1 diabetes (aHR 1.29, 95% CI 1.01, 1.65, based on 67 cases of type 1 diabetes registered after pandemic influenza among unvaccinated people).

## Discussion

In this nationwide study of all residents in Norway below 30 years of age, we found a twofold higher risk of developing type 1 diabetes in individuals who had a specialist care diagnosis or a laboratory-confirmed infection with pandemic influenza.

This is the first study using national registries to address the long-term risk of type 1 diabetes after a pandemic influenza diagnosis. A main strength of the study is the large sample size and the complete inclusion of all residents in Norway. Nearly all persons with type 1 diabetes are diagnosed and treated in the public health system in Norway, and consultations and medications are free for children with type 1 diabetes until 16 years of age. Dispensing of insulin registered in the Norwegian Prescription Database is likely to detect nearly all cases of type 1 diabetes [26], and our algorithm for diagnosis of type 1 diabetes combining registers is likely to ensure true type 1 diabetes in the vast majority of cases [27]. By restricting the study population to those below 30 years of age, we reduced the risk of misclassifying type 2 diabetes as type 1 diabetes. However, as misclassification of diabetes may have occurred in a small number of cases, we performed sensitivity analyses in which we restricted our sample to those below 15 years of age. In this age group, such misclassification is highly unlikely [27].

The main limitation of this study is that we did not capture all cases of influenza, as we only have data on those who sought healthcare and received an influenza diagnosis. It is probable that many people infected with influenza did not seek healthcare, especially those with milder illness. Furthermore, males and females, or different age groups, might seek medical help following different patterns. Administration of antivirals without prescription during the pandemic may also have contributed to fewer people visiting a physician for treatment of influenza infection [28]. Probably those with more severe symptoms were more likely to seek healthcare [29], and our estimates are therefore likely to relate to more severe influenza infections. Infection severity or host response could be important, as influenza diagnosed in primary care did not show a clear association. A possible explanation is that this group contains misclassified cases that might not have been infected with influenza, or that susceptible individuals had more severe symptoms. In our study population, 3% were diagnosed with influenza during the pandemic. Likewise, a report from Sweden suggested that around 6% of the population was diagnosed with H1N1 [30], an estimate probably restricted to those with symptomatic infection. It has been estimated that around 20–30% of the Norwegian population were infected during the 2009–2010 pandemic [31]. Serum A H1N1 antibody positivity can occur after clinical influenza, after mild/quiescent non-clinical influenza or after vaccination with Pandemrix, making it difficult to estimate the true proportion affected by clinical H1N1 infection in a population. A study from Norway conducted in January 2010 showed influenza A (H1N1) serum antibody positivity in up to 65% of younger age groups [32]. Many of these are probably positive because of vaccination with Pandemrix, as around 40% of the total population were vaccinated during the pandemic.

Most individuals were not tested for pandemic influenza as the capacity in laboratories was stretched, and at the point when the pandemic strain was considered to be the primary cause of influenza-like illness, it was regarded as unnecessary to test individuals in primary care when making a diagnosis. Therefore, we do not have laboratory confirmation for the majority of the pandemic influenza diagnoses in our study. During the pandemic period in Norway, there were periods in which non-influenza viruses may have given influenza-like symptoms and resulted in an influenza diagnosis. However, when restricting the exposure period to the pandemic peak period (October to December 2009), when no other influenza strains were circulating and most individuals under age 30 with influenza symptoms were likely to have pandemic influenza, we found similar results.

Previous studies of influenza and type 1 diabetes have been limited to a few small retrospective studies, showing a temporal relationship between H1N1 influenza infection and increased type 1 diabetes incidence [17, 18]. In one study, influenza A antibodies were not associated with initiation of islet autoimmunity in children, but this study did not investigate pandemic influenza A (H1N1), included asymptomatic influenza infections and did not study clinical type 1 diabetes as outcome [33]. In theory, viral infections may affect the progression from islet autoimmunity to clinical diabetes in the small proportion of individuals who are positive for islet autoantibodies [34].

Any association with type 1 diabetes could in theory be due to non-specific immunological mechanisms associated with infections. Respiratory infections in early life and type 1 diabetes have been linked [4–8], and we can consider influenza-like illness as a respiratory viral infection caused by influenza or other viruses. These, and other common viruses causing infection with fever, could be important as cytokine inducer and T cell activators [3]. Our finding that pandemic influenza diagnosis in specialist healthcare was more strongly associated with type 1 diabetes may possibly indicate that an association with type 1 diabetes is stronger with severe infections (needing hospitalisation or other type of specialist care). It is plausible that those with more severe illness had their infection confirmed by laboratory test. Also, it may be that individuals with preclinical diabetes have an underlying higher risk of developing severe influenza. It is likely that most individuals with type 1 diabetes experience a short period of hyperglycaemia before clinical diagnosis of diabetes. The duration of this period is unlikely to last for much more than a few months [35], a period shorter than the average time between influenza and diagnosis of type 1 diabetes in our current analysis. The increased risk of diabetes ascertained from laboratory-confirmed cases could also have occurred by chance. Even though we have a very large dataset, the actual numbers of laboratory-confirmed cases were small.

The dominating circulating influenza types usually differ by season, and it is difficult to discern from our data whether any specific strain tends to be more strongly associated with type 1 diabetes. We found the strongest evidence for pandemic influenza. However, there was also increased risk of type 1 diabetes after influenza in the following season (2010–2011), where influenza A (H1N1)pdm09 virus and influenza B co-dominated (albeit with slightly more influenza B) [36]. It is also possible that the 2009 pandemic influenza strain has stronger tropism for pancreatic cells than other influenza strains [37]. The immunological response to influenza infection with different severity and with different virus strains may differ and is not well understood [38]. More studies are needed to conclude on the role of different seasonal influenza viruses in type 1 diabetes aetiology.

Our large register cohort does not include information on pre-diagnostic diabetes associated autoantibodies. Hence, we could not investigate whether influenza infections induced or accelerated autoimmunity. Viruses could contribute to the development of clinical diabetes through stress and inflammation in individuals with autoimmunity (non-specific effect of virus infections) [39].

We could speculate that preventing viral infections, for example through influenza vaccination, could help reduce the incidence of type 1 diabetes. In a recent paper from The Environmental Determinants of Diabetes in the Young (TEDDY) study, the Pandemrix vaccination was associated with a lower risk of islet autoimmunity in children at increased genetic risk in Finland, whereas no difference was seen in Sweden [40]. Unfortunately, we do not have access to data on autoimmunity in our register-based study. In line with the Swedish data we did not find any association with Pandemrix and type 1 diabetes in our study [20]. In Norway, many of those who were vaccinated with Pandemrix had already been infected, or were infected with influenza A (H1N1) after vaccination, but before effective protective antibodies had been induced [21].

In conclusion, we could not demonstrate a clear association between clinically reported pandemic influenza infection and incident type 1 diabetes in this register-based cohort study. We did, however, find a twofold excess of incident diabetes in the subgroups with laboratory-confirmed pandemic influenza A (H1N1) or pandemic influenza diagnosed in specialist healthcare. This suggests that respiratory infections may play a role in the aetiology of type 1 diabetes, but more studies are warranted.

## Electronic supplementary material

ESM (PDF 909 kb)

## Data Availability

The datasets generated during and/or analysed during the current study are not publicly available because of data protection regulations.

## Abbreviations

ICPC-2: International Classification of Primary Care, Second Edition
MSIS: Norwegian Surveillance System for Communicable Diseases
