# Supplementary material for: Pandemic influenza and subsequent risk of type 1 diabetes: a nationwide cohort study
Source: Diabetologia. 2018 Jun 22;61(9):1996–2004. doi: 10.1007/s00125-018-4662-7 (PMC6096634; doi:10.1007/s00125-018-4662-7)
Supplement: Supplementary file 1 — (PDF 909 kb) [file 125_2018_4662_MOESM1_ESM.pdf]

Electronic Supplementary Material (ESM)

Influenza diagnosis and subsequent risk of type 1 diabetes – a nationwide cohort study

ESM Methods

Details of the Norwegian health registries and the influenza infection.

The Norwegian Prescription Database (NorPD) records all drugs dispensed to individual patients from pharmacies in Norway from 2004, using the Anatomical Therapeutic Chemical Classification (ACT). The Norwegian Patients Registry (NPR) was established in 2008 and, at individual level, includes both inpatients and outpatients using the International Classification of Diseases, version 10 (ICD-10 codes). Diagnoses from the Primary Care Database (KUHR) were provided from Norwegian Health Economics Administration (HELFO), and consist of electronic remuneration claims, from 2006 onwards, using the International Classification of Primary Care, Second edition (ICPC-2 codes) (ESM Table 1). Reporting to these independent nation-wide databases is mandatory, and, for NPR and KUHR, required to receive governmental reimbursement. Laboratory confirmed influenza cases were, during the 2009-2010 pandemic, registered at the nationwide system for surveillance of infectious diseases (MSIS). Data on seasonal lab-confirmed influenza are not reported at individual level to MSIS.

From Statistics Norway we obtained data on educational level and place of birth. We have dates of birth, death, emigration and immigration from the National Registry.

ESM Tables

ESM Table 1. List of codes used to identify type 1 diabetes cases and influenza diagnosis. ICD-10 codes: International Classification of Diseases, version 10. ACT: Anatomical Therapeutic Chemical Classification. ICPC-2: International Classification of Primary Care, Second edition.

Type 1 diabetes		
**Norwegian Patient Registry (NPR)**	**The Norwegian Prescription Database (NorPD)**	**Primary healthcare database (KUHR)**
Diagnostic ICD-10 codes<br><br>E10	Medications ATC codes<br><br>A10A, A10B	Diagnostic ICPC-2 codes<br><br>T89
**Influenza diagnosis**		
**Norwegian Patient Registry (NPR)**	**Primary healthcare database (KUHR)**	**Surveillance System for Communicable Diseases (MSIS)**
Diagnostic ICD-10 codes<br><br>J09, J10, J11	Diagnostic ICPC-2 codes<br><br>R80 Influenza	Date lab test for influenza A (H1N1)pdm09

ESM Table 2. Characteristics of the study population < 30 years of age included in the seasonal influenza analysis.

Characteristics	All subjects	Influenza 2006	Influenza 2006/07	Influenza 2007/08	Influenza 2008/09	Influenza 2010/11	Influenza 2011/12	Influenza 2012/13
n	2,512,674	25,090	32,299	19,691	22,840	36,763	27,397	39,179
**Sex**								
Male	1,282,780 (51)	12,489 (50)	15,587 (48)	9,722 (49)	10,950 (48)	17,667 (48)	12,919 (47)	18,795 (48)
Female	1,229,894 (49)	12,601 (50)	16,712 (52)	9,969 (51)	11,890 (52)	19,096 (52)	14,478 (53)	20,384 (52)
**Year of birth**								
1975-1979	240,002 (9)	3,764 (15)	5,577 (17)	2,449 (12)	1,391 (6)	-	-	-
1980-1989	691,255 (27)	9,629 (38)	15,062 (47)	10,530 (53)	12,622 (55)	15,675 (43)	10,841 (40)	13,199 (34)
1990-1999	669,192 (27)	7,650 (30)	5,831 (18)	3,384 (17)	4,583 (20)	11,524 (31)	8,501 (31)	13,184 (34)
2000-2009	633,342 (25)	4,047 (16)	5,829 (18)	3,328 (17)	4,244 (19)	8,958 (24)	6,436 (23)	9,037 (23)
>2010	278,883 (11)	-	-	-	-	606 (2)	1,619 (6)	3,759 (10)
**Education level** [a]								
≤10 years	216,172 (9)	2,001 (8)	2,818 (9)	1,849 (9)	2,142(9)	3,510 (10)	2,642 (10)	4,027 (10)
11-13 years	856,496 (34)	10,746 (43)	13,626 (42)	8,394 (42)	9,619 (42)	15,480 (42)	11,183 (41)	15,968 (41)
≥14 years	1,333,044 (53)	12,199 (48)	15,574 (48)	9,198 (47)	10,702 (47)	17,177 (47)	13,070 (48)	18,366 (47)
No information	106,962 (4)	144 (1)	281 (1)	250 (1)	377 (2)	596 (2)	502 (2)	818 (2)
**Place of birth**								
Norway	2,155,907 (86)	22,998 (92)	29,098 (90)	17,390 (88)	20,019 (88)	32,157 (87)	24,049 (88)	34,265 (87)
Europe (except Norway)	188,144 (7)	852 (3)	1,314 (4)	977 (5)	1,218 (5)	2,130 (6)	1,700 (6)	2,468 (6)
Outside Europe	168,623 (7)	1,240 (5)	1,887 (6)	1,324 (7)	1,603 (7)	2,476 (7)	1,648 (6)	2,446 (6)

[a] **Education level:** The highest education level the individual have achieved up to 2013 or the highest attained education level of their parents.

ESM Figures

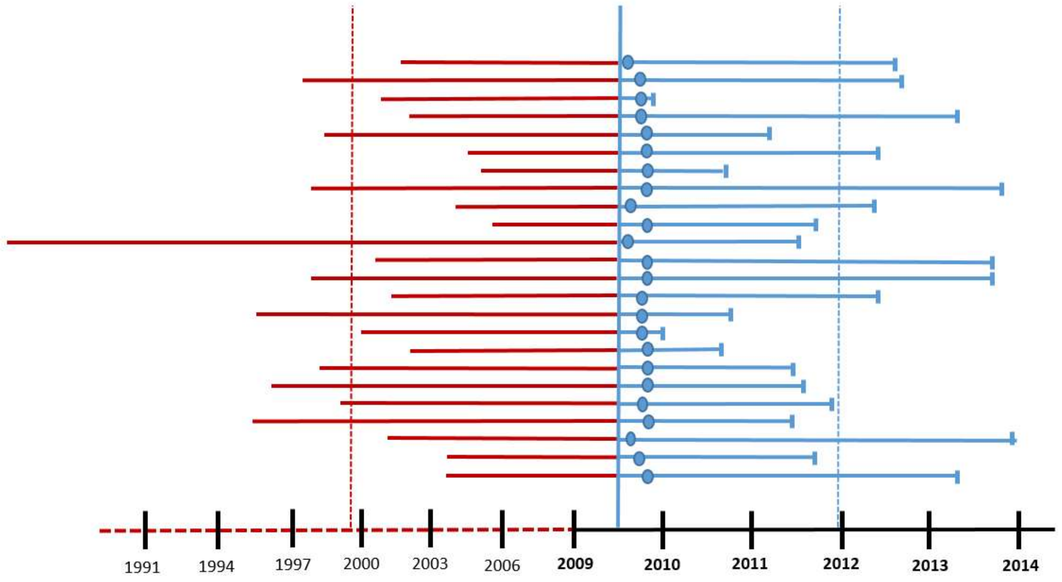

ESM Fig. 1. Time of H1N1 lab-confirmed cases and subsequent diagnosis of type 1 diabetes in 24 exposed cases. On the left hand side in red: time from birth until study start. On the right hand side in blue: time from start of follow-up to diagnosis of type 1 diabetes. Blue circle represent time for H1N1 lab positive test. The average age at H1N1 positive test was 10 years, and the average time from H1N1 to type 1 diabetes diagnosis was 2.2 years.

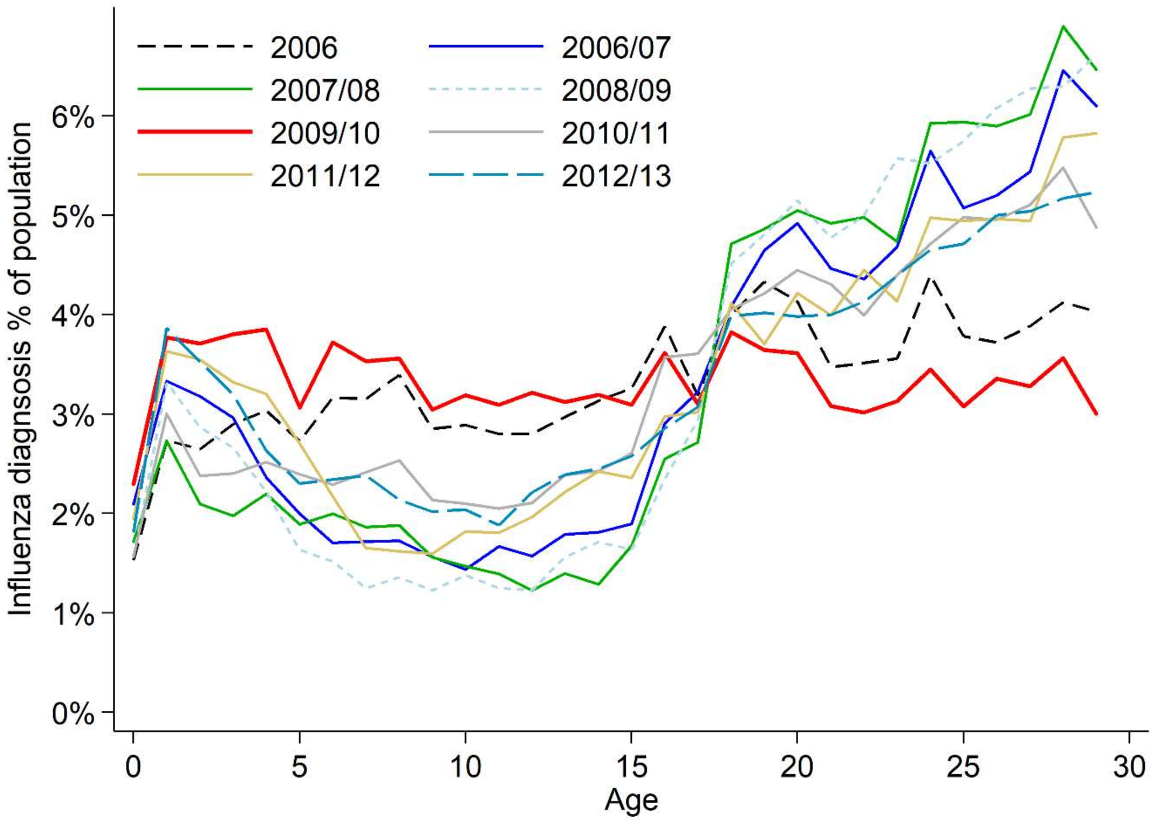

ESM Fig. 2. Percent of the population diagnosed with influenza by age and season

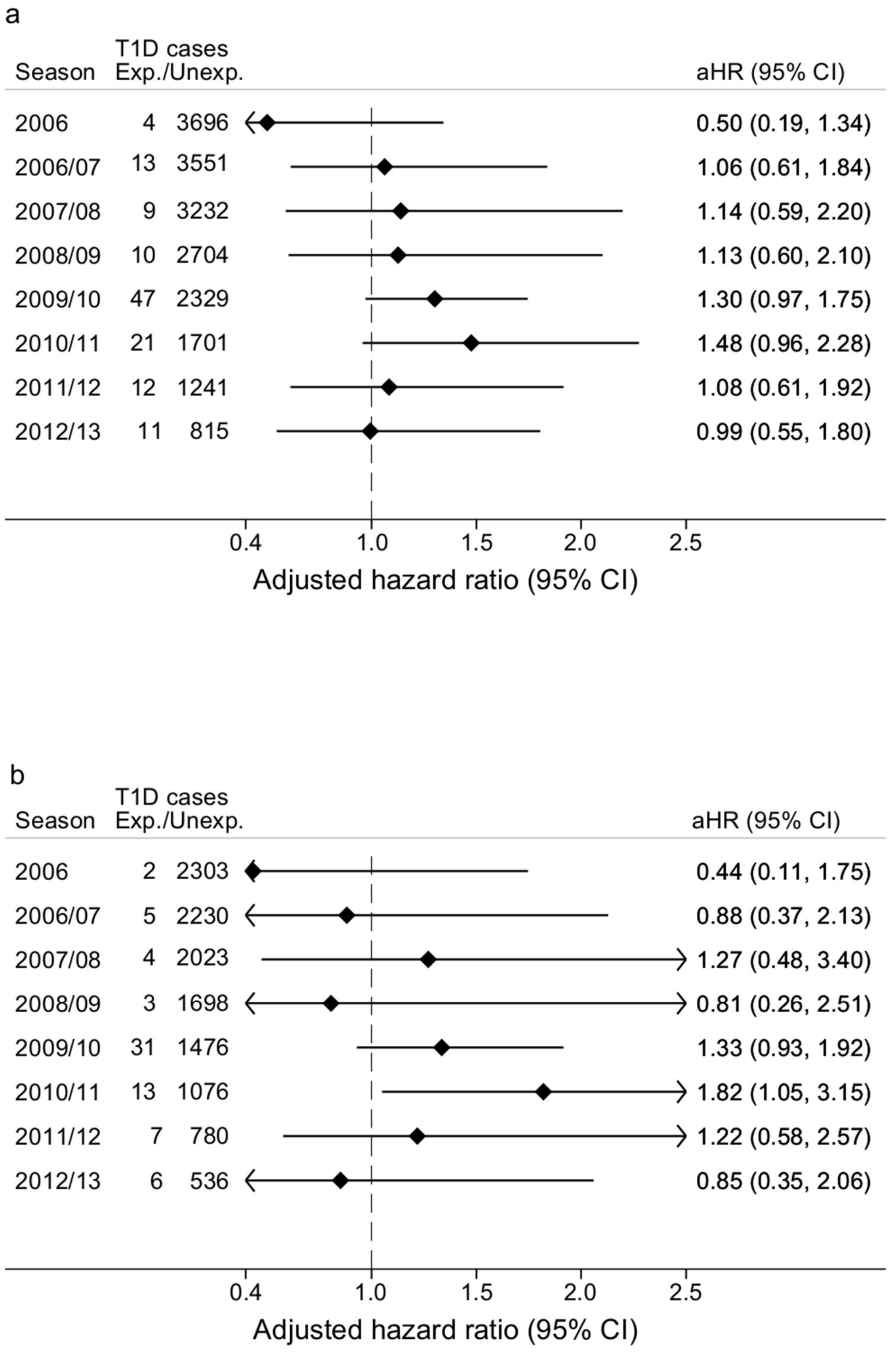

ESM Fig. 3. Association between seasonal influenza diagnosis and risk of type 1 diabetes, when limiting the exposure period to the first 2 years after infection. a) Follow-up to 30 years of age, b) Follow-up to 15 years of age Hazard ratios were adjusted for year of birth, sex, place of birth, education and pandemic influenza vaccination. ILI, Influenza Like Illness. Follow up time for seasonal influenza from 1st January 2006 to 30th June 2014 (the pandemic season, 2009-2010, shows the same data as in figure 2). Exp., Exposed; Unexp., Unexposed.
